# Gun Carrying Among Military-Connected Youth With Past-Year Suicidal Ideation and Suicide Plans

**DOI:** 10.1001/jamanetworkopen.2024.24916

**Published:** 2024-07-31

**Authors:** Ian H. Stanley, Ian F. Eisenhauer, Ashley Brooks-Russell, Eric J. Sigel

**Affiliations:** 1 Department of Emergency Medicine, University of Colorado School of Medicine, Anschutz Medical Campus, Aurora; 2 Center for COMBAT Research, University of Colorado School of Medicine, Anschutz Medical Campus, Aurora; 3 Firearm Injury Prevention Initiative, University of Colorado Anschutz Medical Campus, Aurora; 4 Injury and Violence Prevention Center, University of Colorado Anschutz Medical Campus, Aurora; 5 Department of Pediatrics, University of Colorado School of Medicine, Anschutz Medical Campus, Aurora

## Abstract

This cross-sectional study compares gun carrying practices and participation in violence prevention programs among youth with vs without a parent in the US military.

## Introduction

Suicide prevention among military service members and their families, including children, is a key priority of the US Department of Defense.^1^ Firearm suicide is a leading cause of death among youth; firearm access broadly,^2^ and gun carrying specifically,^3^ is associated with an increased risk of suicide. Participation in violence prevention programs may decrease risk. State-level data indicate elevated levels of gun carrying among military-connected youth, perhaps due to greater firearm ownership in military families.^4^ Using nationally representative data, we examined differential handgun-carrying practices and participation in violence prevention programs between youths with a parent in the military and those without. Extending prior work, we examined handgun carrying among youth reporting suicidal ideation or suicide plans in the past year because handgun carrying among these individuals may be associated with increased risk for lethal outcomes.^5^

## Methods

In this cross-sectional study, we analyzed data from the 2021 National Survey on Drug Use and Health (NSDUH),^6^ a representative survey of noninstitutionalized US civilians aged 12 years or older. Data were collected from January 14, 2021, through December 20, 2021. This analysis was approved by the Colorado Multiple Institutional Review Board. Youths and their parents provided verbal informed consent. We followed the STROBE reporting guideline.

We analyzed responses to the specific survey items from youth aged 12 to 17 years (eTable in Supplement 1) . We used logistic regression analyses to examine the association between having a parent in the military (1, yes; 0, no) and past-year handgun carrying (1, yes; 0, no), controlling for age, sex, race, and ethnicity. We used listwise deletion and sampling weights provided by NSDUH.^6^ Two-sided *P* < .05 was significant, and SPSS, version 29.0 (IBM Corp) was used for analyses.

## Results

This study included 10 045 youths (49.0% female and 51.0% male) (Table 1); 3.2% reported a military connection and 4.0% reported handgun carrying in the past year (3.8% military-connected youth vs 4.0% non–military-connected youth). Overall, 15.4% of youth reported having suicidal ideation within the past year and 6.6% reported having suicide plans. In the past year, military-connected youth compared with their counterparts had higher odds of having suicidal ideation (18.1% vs 15.3%; AOR, 1.36 [95% CI, 1.35-1.37]) and suicide plans (10.7% vs 6.5%; AOR, 1.92 [95% CI, 1.90-1.93]) (Table 2). Among youth reporting suicidal ideation or suicide plans in the past year, military- vs non–military-connected youth had lower odds of reporting handgun carrying in the past year (AOR, 0.74 [95% CI, 0.72-0.76] and AOR, 0.24 [95% CI, 0.22-0.25], respectively). Military- vs non–military-connected youth had higher odds of having participated in a violence prevention program in the past year (15.8% vs 8.1%; AOR, 2.02 [95% CI, 2.01-2.03]).

**Table 1.** Participant Demographic Characteristics^a^

Characteristic	Unweighted No. (weighted %)		
	Overall sample (N = 10 045)^b^	Military-connected youth (n = 398)	Non–military-connected youth (n = 9647)
Age category			
12-13 y	3327 (33.6%)	155 (38.4%)	3172 (33.5%)
14-15 y	3367 (32.5%)	132 (37.1%)	3235 (32.3%)
16-17 y	3351 (33.9%)	111 (24.5%)	3240 (34.2%)
Sex			
Female	4945 (49.0%)	179 (41.2%)	4766 (49.2%)
Male	5100 (51.0%)	219 (58.8%)	4881 (50.8%)
Race and ethnicity^c^			
American Indian or Alaska Native	105 (0.7%)	6 (0.5%)	99 (0.7%)
Asian	477 (5.7%)	6 (1.3%)	471 (5.9%)
Black or African American	1370 (13.4%)	57 (12.5%)	1313 (13.5%)
Hispanic	2262 (24.7%)	82 (22.5%)	2180 (24.8%)
Multiracial	621 (3.5%)	26 (2.6%)	595 (3.5%)
Native Hawaiian or Other Pacific Islander	35 (0.5%)	0 (0.0%)	35 (0.5%)
White	5175 (51.5%)	221 (60.6%)	4954 (51.2%)

^a^ All χ^2^ significant at *P* < .001.

^b^ There were 10 743 youth aged 12 to 17 years in the 2021 National Survey on Drug Use and Health dataset. Data on parental military status were missing for 698 of these youths; thus, the total analytic sample was 10 045 youths.

^c^ Self-reported race and ethnicity data were analyzed due to differences in firearm carrying across these characteristics.

**Table 2.** Past-Year Characteristics for Youth With vs Without a Parent in the Military^a^

Past-year characteristic	Weighted %			Age-, sex-, and race/ethnicity-adjusted comparisons of military-connected and non–military-connected youth	
	Overall sample	Military-connected youth	Non–military-connected youth	AOR (95% CI)	*P* value
Handgun carrying					
Among overall sample	4.0	3.8	4.0	0.92 (0.91-0.93)	<.001
Among youth with suicidal ideation	5.2	4.0	5.2	0.74 (0.72-0.76)	<.001
Among youth with suicide plans	7.6	1.9	7.9	0.24 (0.22-0.25)	<.001
Suicidal experiences					
Suicidal ideation	15.4	18.1	15.3	1.36 (1.35-1.37)	<.001
Suicide plans	6.6	10.7	6.5	1.92 (1.90-1.93)	<.001
Participated in a violence prevention program	8.3	15.8	8.1	2.02 (2.01-2.03)	<.001

^a^ The logistic regression analysis was adjusted for age categories, sex, and race and ethnicity. The 2021 National Survey on Drug Use and Health was the source of the data.

## Discussion

In this cross-sectional study, we found that military-connected youth with suicidal ideation or suicide plans in the past year had lower odds of reporting handgun carrying in the past year. Risk and harm reduction efforts aimed at service members are common and military service wide, but similar programs are not standard for military dependents.^1^ Nonetheless, military service–wide efforts to create community, reduce stigma, and promote lethal means safety specifically aimed at suicide prevention may have secondary benefits for dependent youth. This includes registration requirements for firearms stored on a military base that specifically addresses child safety, including lock mandates and secure storage options.

Study limitations include a lack of information on whether youths currently live with their parent, nature of the handgun carried (eg, parent owned, reason), and type of military connection (eg, Active Component, Reserve, National Guard). Findings of this study suggest that children who report mental health challenges and have a parent in the military may have a lower risk of handgun carrying behaviors. Future research identifying factors of this association, such as service member–directed prevention efforts or youth-directed violence prevention efforts, may inform development and sustainment of effective firearm injury prevention efforts among military- and non–military-connected youth.

## Notes

Supplement 1. **eTable.** Survey Items and Responses

Supplement 2.
Data Sharing Statement
