# Supplementary material for: Gun Carrying Among Military-Connected Youth With Past-Year Suicidal Ideation and Suicide Plans
Source: JAMA Netw Open. 2024 Jul 31;7(7):e2424916. doi: 10.1001/jamanetworkopen.2024.24916 (PMC11292444; doi:10.1001/jamanetworkopen.2024.24916)
Supplement: Supplement 1. — eTable. Survey Items and Responses [file jamanetwopen-e2424916-s001.pdf]

Supplemental Online Content

Stanley IH, Eisenhauer IF, Brooks-Russell A, Sigel EJ. Gun carrying among military-connected youth with past-year suicidal ideation and suicide plans. *JAMA Netw Open*. 2024;7(7):e2424916. doi:10.1001/jamanetworkopen.2024.24916

eTable. Survey Items and Responses

This supplemental material has been provided by the authors to give readers additional information about their work.

© 2024 Stanley IH et al. *JAMA Network Open*.

eTable. Survey Items and Responses

Construct	Survey Item	Survey Response Options	Coding
Parental Military Status	Participants were instructed to consider specific family members (e.g., father, mother) “whether or not they live with you” and “whether they are biological, step or adoptive” and then asked, “Are any of these family members currently serving in the United States military?”	• Yes<br>• No	Respondents who stated “Yes” to their father or mother being in the military were coded as military-connected.
Handgun Carrying (Past-Year)	“During the past 12 months, how many times have you carried a handgun?”	• 0 times<br>• 1 or 2 times<br>• 3 to 5 times<br>• 6 to 9 times<br>• 10 or more times	Any non-zero response was coded as positive for past-year handgun carrying.
Suicidal Ideation (Past-Year)	“At any time in the past 12 months, that is from [DATEFILL] up to and including today, did you seriously think about trying to kill yourself?”	• Yes<br>• No	Respondents who reported “Yes” were coded as positive for past-year suicidal ideation.
Suicide Plan (Past-Year)	“During the past 12 months, did you make any plans to kill yourself?”	• Yes<br>• No	Respondents who reported “Yes” were coded as positive for past-year suicide plans.
Participation in Violence Prevention Program (Past-Year)	“During the past 12 months have you participated in a violence prevention program, where you learn ways to avoid fights and control anger?”	• Yes<br>• No	Respondents who reported “Yes” were coded as positive for past-year participation in a violence prevention program.

© 2024 Stanley IH et al. *JAMA Network Open*.
