# Supplementary material for: Gun Carrying Among Military-Connected Youth With Past-Year Suicidal Ideation and Suicide Plans
Source: JAMA Netw Open. 2024 Jul 31;7(7):e2424916. doi: 10.1001/jamanetworkopen.2024.24916 (PMC11292444; doi:10.1001/jamanetworkopen.2024.24916)
Supplement: Supplement 2. — Data Sharing Statement [file jamanetwopen-e2424916-s002.pdf]

Data Sharing Statement

Stanley. Gun Carrying Among Military-Connected Youth With Past-Year Suicidal Ideation and Suicide Plans. *JAMA Netw Open*. Published July 31, 2024.
doi:10.1001/jamanetworkopen.2024.24916

Data
Data available: No

Additional Information
Explanation for why data not available: The data are publically available from the Substance Abuse and Mental Health Services Administration (SAMHSA).
